# The role of the practice order: A systematic review about contextual interference in children

**DOI:** 10.1371/journal.pone.0209979

**Published:** 2019-01-22

**Authors:** Judith V. Graser, Caroline H. G. Bastiaenen, Hubertus J. A. van Hedel

**Affiliations:** 1 Paediatric Rehab Research Group, Rehabilitation Centre for Children and Adolescents, University Children’s Hospital Zurich, Affoltern am Albis, Switzerland; 2 Children’s Research Centre CRC, University Children’s Hospital Zurich, Zurich, Switzerland; 3 Research Line Functioning and Rehabilitation CAPHRI, Department of Epidemiology, Maastricht University, Maastricht, the Netherlands; Kennedy Krieger Institute/Johns Hopkins Medicine, UNITED STATES

## Abstract

**Aim:**

We aimed to identify and evaluate the quality and evidence of the motor learning literature about intervention studies regarding the contextual interference (CI) effect (blocked vs. random practice order) in children with brain lesions and typically developing (TD) children.

**Method:**

Eight databases (Cinahl, Cochrane, Embase, PubMed, Pedro, PsycINFO, Scopus and Web of Knowledge) were searched systematically with predefined search terms. Controlled studies examining the CI effect in children (with brain lesions or TD) were included. Evidence level, conduct quality, and risk of bias were evaluated by two authors independently. A best evidence synthesis was performed.

**Results:**

Twenty-five papers evaluating TD children were included. One of these studies also assessed children with cerebral palsy. Evidence levels were I, II, or III. Conduct quality was low and the risk of bias high, due to methodological issues in the study designs or poor description thereof. Best evidence synthesis showed mainly no or conflicting evidence. Single tasks showed limited to moderate evidence supporting the CI effect in TD children.

**Conclusion:**

There is a severe limitation of good-quality evidence about the CI effect in children who practice different tasks in one session, especially in children with brain lesions.

## Introduction

Children with brain lesions, such as cerebral palsy (CP), frequently have to deal with impairments of the sensorimotor system, leading to restrictions in activities and independence which could affect participation in daily life [1]. Intensive therapeutic interventions are needed to address these limitations. Usually, several tasks or skills are practiced during single therapeutic sessions to cover a broad range of impairments and limitations and to keep the children engaged. However, learning one skill can be influenced by practicing another one during the same session [2]. This so-called contextual interference effect [3] has been established by Battig and has been described later in various motor learning studies. These studies showed that the contextual interference effect was low when different tasks are practiced in a blocked order, meaning that one task is practiced until it is learned before moving to the next [4]. A high contextual interference effect is achieved if different tasks are practiced in a random order [4]. Most evidence about the contextual interference effect has been obtained in healthy young adults with the intent of improving practice schedules in sports. In this population, a low contextual interference effect results in better acquisition but worse transfer and retention of task performance. The findings are the opposite if practicing with high contextual interference [4,5].

For paediatric patients after rehabilitation discharge, it is important that learned tasks can be retained over time and generalised to other conditions or tasks. The evidence is lacking, though, whether this specific population would also benefit from a high contextual interference, and whether this can be achieved by practicing in a random order.

Several aspects seem to influence the contextual interference effect. Magill and Hall mentioned that task characteristics (e.g. non-laboratory tasks such as beanbag throwing vs. laboratory tasks such as coincident anticipation timing tasks) and subject characteristics like age or the level of experience are important, although it remains unclear how age exactly influences the contextual interference effect [5]. Therefore, it remains unclear what the optimal practice order in typically developing children (e.g., [6–8]) and in children with brain lesions undergoing neurorehabilitation is.

Some reviews about contextual interference exist [5,9–12], but none of them included a systematic evaluation regarding the effects and quality of intervention studies in the field of paediatric motor learning, which limits the relevance for the field of paediatric neurorehabilitation. This shortage of knowledge is unfortunate since neurorehabilitation is based on motor learning principles [13], and therapeutic interventions could be improved by adhering to such principles [14]. As we assume that results from contextual interference studies involving typically developing children could be better generalised to children with brain lesions compared to results obtained from healthy adults, the objective of this systematic review was to investigate the evidence of contextual interference in children with congenital or acquired brain lesions and typically developing children. The research question is the following: What is the evidence concerning the contextual interference effect for children with congenital or acquired brain injuries and typically developing children?

## Methods

This review was conducted by following certain aspects of the guidelines provided by the American Academy of Cerebral Palsy and Developmental Medicine (AACPDM) [15] and supplemented by the risk of bias tool provided by the Cochrane Collaboration [16]. The procedure is described in detail below.

Since no participants were required for this study, obtaining ethical approval was not necessary.

### Inclusion and exclusion criteria

We defined inclusion criteria in line with PICO (**P**opulation, **I**ntervention, **C**ontrol, **O**utcome) and included studies assessing children (with congenital or acquired brain injuries and/or typically developing) in the age range between 1 and 18 years (Population). We included motor learning studies examining the contextual interference effect with a random practice order group (Intervention) and at least one blocked practice order group (Control). Any outcome evaluating the acquisition, retention, and/or transfer of the learned skill (Outcome) was considered selectable. JG defined the search terms based on PICO and HVH reviewed the search terms. The following search terms were used:

**Population:** ‘child’, ‘children’, ‘childhood’, ‘paediatrics’, ‘adolescents’, ‘adolescence’, ‘youths’, ‘student’, ‘elementary’, ‘high school’.

**Intervention:** ‘motor learning’, ‘skill learning’, ‘contextual interference’, ‘practice order’;

**Control group:** ‘blocked and random’;

**Outcome:** ‘performance’, ‘acquisition’, ‘retention’, ‘transfer’, ‘generalisation’, and ‘generalisability’.

Search terms were customised for each database including the use of MESH terms when applicable. We refrained from adding methodological criteria (e.g. randomisation procedures for group allocation) to get a broad overview of the existing literature. The search was performed by the first author on the databases Cinahl, Cochrane, Embase, PubMed, Pedro, PsycINFO, Scopus and Web of Knowledge (an example of a detailed search strategy is shown in S1 Table). The reference lists of original research papers and systematic reviews were screened for further eligible studies. The primary search was performed in March 2015 (for the period 1960 to March 2015) and updated in December 2016 (period 2015 to 2016).

We excluded studies that allowed a true practice phase (i.e., not a typical familiarization phase which normally consists of a few trials that are performed to have the participant give an idea about the skill to be learned) before the actual acquisition phase. There is a phenomenon called “learning to learn” [17] which describes the beneficial influence of prior practice experience on an unfamiliar motor task [18]. In humans, this phenomenon has been observed in visuomotor [18,19], and cognitive tasks [20–22]. In a recent study with healthy young adults practicing a dynamic balance task, the “learning to learn” phenomenon could not be reproduced [23]. We included studies with a wide variety of motor tasks. Since there is no general accordance about the “learning to learn” phenomenon we decided to exclude studies with prior practice phase because this would affect the comparability with studies without such a practice phase.

We also excluded conference papers, studies of which only the abstracts were available, unpublished dissertations, and studies in a language other than English or German.

### Selection procedure

Firstly, JG and HVH read the titles and abstracts and decided upon eligibility independently. Secondly, the same authors read the full texts of the papers that were considered eligible and decided on final eligibility independently. In cases of disagreement, the authors discussed until consensus was reached.

### Data extraction and analysis

JG summarised relevant data using a standardised data extraction sheet. Included were the type of study, participants (population, age, number per group), task, information regarding the acquisition, retention and transfer phases, including time points, duration, used outcome measures (e.g. anticipation timing task) and parameters (e.g. variable and random error), as well as the results. In case of incomplete reporting of patient characteristics or study procedure, we contacted the authors of the original publication.

We had planned to pool data when studies were comparable regarding populations, interventions, outcomes, and types of studies. If we were not able to follow this approach, due to heterogeneity of the studies, pooling within relevant subgroups was considered. When we would choose to refrain from pooling completely, because meaningful subgroups could not be built a best evidence synthesis would be performed using the levels of evidence described by Tulder et al. [24]. The results of each study would be rated as significant (favouring blocked or random order), inconsistent or not significant. Consistency of the results within one study would be given if 75% of the comparisons (e.g. measures, parameters, tasks) would provide similar results (e.g. random was better than blocked for the retention). Then the evidence of the different tasks (several studies per task, if possible) was rated according to the suggestions by Tulder et al. [24]: strong (consistent findings among multiple high quality randomised controlled trials (RCTs)), moderate (consistent findings among multiple low quality RCTs and/or controlled clinical trials (CCTs) and/or high one high quality RCT), limited (one low quality RCT and/or CCT, conflicting (inconsistent findings among multiple RCTs and/or CCTs; inconsistent findings among different parameters within one trial (if only one trial is available) or no evidence from trials (no RCTs or CCTs). Consistency of the studies assessing similar tasks would be given if more than 75% of the studies showed results in the same direction.

### Methodological quality assessment

JG and CB assessed the level of evidence and the methodological quality of the eligible studies independently from each other, as recommended by the AACPDM [15]. The detailed descriptions of the evidence levels are displayed in S2 Table. The evaluation of the methodological quality included the seven aspects also described by the AACPDM [15] (for details see S3 Table).

We also evaluated the risk of bias. Bias is defined as any systematic error that results in an incorrect estimate of the true effect of an exposure on the outcome of interest. Bias can result in an over- or underestimation of the true value depending on the type of bias. We considered selection bias (i.e. sequence generation, allocation concealment), performance bias (i.e. blinding participants, personnel), attrition bias (i.e. incomplete outcome data), reporting bias (i.e. selective reporting), and other sources of bias (see also S4 Table). As bias is a potential threat to the trustworthiness of study results, the strength of a conclusion of a systematic review should be adjusted accordingly [16].

JG and CB rated the risk of bias according to recommendations described in the Cochrane Handbook for Systematic reviews of Interventions [16]. Discrepancies between the two authors were discussed until consensus was reached.

## Results

### Search results

**Fig 1:**
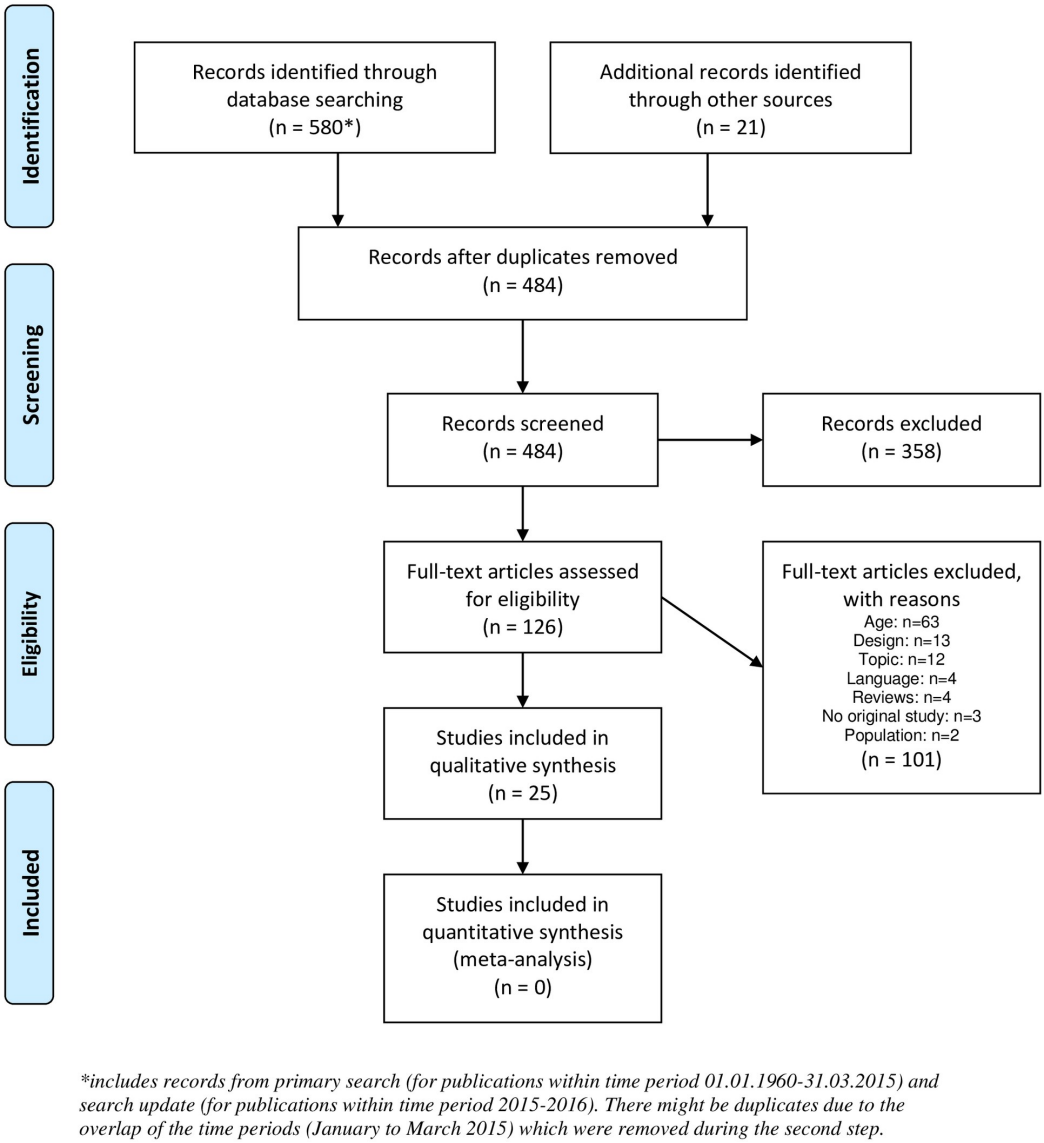
Flowchart of the search process. Flowchart of the primary search (time period between 01.01.1960 and 31.03.2015) the updated search (time period of 2015 and 2016), and the inclusion and exclusion process. *From*: Moher D, Liberati A, Tetzlaff J, Altman DG, The PRISMA Group (2009). *P*referred *R*eporting *I*tems for *S*ystematic Reviews and *M*eta-*A*nalyses: The PRISMA Statement. PLoS Med 6(7): e1000097. doi:10.1371/journal.pmed1000097. For more information, visit www.prisma-statement.org.

The primary search in the databases led to 503 records (Fig 1). The 11 full texts that were excluded due to topic reasons were not motor learning studies or did not evaluate contextual interference. Thirteen full texts were excluded due to design issues (5 had no random order practice group, 4 had several practice orders within the same group (i.e., no parallel study design), 2 had a preparation phase, in which participants were allowed to practice for several sessions prior to the acquisition phase, 1 study had no blocked practice but a series of trials in blocks, and 1 had no blocked group but two random groups with different levels of variation). By checking the references of eight reviews (four were found during the primary search, three within the references of these reviews and one by coincidence on google scholar) that focussed on contextual interference in general (not specifically for children), we could include two additional studies. The references of the original research studies contained no further eligible studies. The updated search resulted in one additional study (Fig 1).

We included 25 papers in this systematic review. One paper presented three different experiments with three different samples [25]. This paper is handled as three separate studies in our review. Four of 27 studies assessed typically developing children as well as participants with disorders: Down’s Syndrome [6], learning disabilities [26], mild mental handicaps [27], and CP [28]. Only the groups of typically developing children (i.e. with no diagnoses or disorders) were included for the best evidence synthesis. Concerning patient groups with congenital or acquired brain lesions, we included only the study in which children with CP learned to grasp unfamiliar objects [28]. Information of each study is presented in Table 1.

**Table 1:** Summary of the included studies.

Study	Description		Acquisition	Retention	Transfer	Results		
	*Participants*	*Task/skill*	*Timeframe*	*Timeframe*	*Timeframe*	*Acquisition*	*Retention*	*Transfer*
	*n total*		*Outcome measure*	*Outcome measure*	*Outcome measure*			
	*n group*		*Outcome parameter*	*Outcome parameter*	*Outcome parameter*			
Bertollo et al., 2010 [29]	Female high school students (mean age = 15.8 yrs., SD = 1.3 yrs.)	Dance step sequence (different sequences)	3 weeks 2 sessions / week (30 min each)	After 21 days	NA	Blocked > random (p < 0.01, d = 0.90, 95% CI = 0.25–1.56)	No significant group difference	NA
	Total: 40		Step sequence	Step sequence	NA			
	Blocked: 20 Random: 20		Score of spatial and temporal accuracy	Score of spatial and temporal accuracy	NA			
Bortoli et al., 1992 [30]	9th grade students (mean age = 14.6. yrs., SD = 0.7 yrs.)	Volleyball skills (bump, volley, surf)	6 weeks 1 session / week	After 1 week	After 1 week	Blocked = random (F < 1.00)	No significant group difference	Long transfer: random > blocked (F_3,48_ = 2.97, p < 0.05, all 4 groups) Short transfer: not reported
	Total: 52		Specified targets	Specified targets	Targets 1 meter (short transfer), 1 meter behind (long transfer)			
	Blocked: 13 Random: 13 Serial Organisation: 13 Serial organisation (very high CI): 13		Scores of accuracy	Scores of accuracy	Scores of accuracy			
Broadbent et al., 2015 [31]	Intermediate—level tennis players (blocked practice group mean age = 12.9 yrs., SD = 1.6 yrs.; random practice group mean age = 13.2 yrs., SD = 1.6 yrs.)	Reaction to simulation: tennis skills (forehand groundstroke, forehand smash, forehand volley)	3 weeks 1 session / week (15 min each)	After 7 days	After 7 days	No main effect for group (F_1,16_ = 0.10, p = 0.76)	No main effect for group (F_1,16_ = 2.9, p = 0.11) Significant group x test interaction (F_1,16_ = 6.03, p = 0.03) random (mean ± SD = 71.7 ± 5.3%) > blocked (mean ± SD = 63.3 ± 6.0%)	Response accuracy: no significant group effect (F_1,14_ = 0.03, p = 0.86) Decision time: significant difference (F_1,14_ = 7.19, p = 0.02) random (mean ± SD = 98 ± 89 ms) > blocked (mean ± SD = 238 ± 118 ms)
	Total: 18		Tasks themselves	Tasks themselves	Tasks themselves			
	Blocked: 9 Rrandom: 9		Response accuracy = primary outcome	Response accuracy = primary outcome	Response accuracy Decision time			
Del Rey et al., 1983 [32]	Children, half of the sample experienced in sports requiring coincident anticipation (median age = 8.33 yrs., range = 6.50 yrs. to 10.67 yrs.)	Anticipation timing task	1 session 60 trials	NA	Immediately after	Significant group difference blocked > random (probably for both, absolute and variable error, since "analyses for the two errors, for the most part, agreed" p. 582)	NA	Absolute error: no significant group difference (p = 0.05) (blocked: mean ± SD = 70 ± 45 ms; random: mean ± SD = 106 ± 62 ms) Variable error: no significant group difference (p = 0.05) (blocked: mean ± SD = 74 ± 25 ms; random: mean ± SD = 98 ± 42 ms)
	Total: 80		Task itself	NA	Task itself, new speed			
	Blocked: 40 Random: 40 *20 girls and boys in each practice group*		Absolute error Variable error	NA	Absolute error Variable error			
Duff et al., 2003 [28]	Children with hemiplegic CP (mean age = 10 yrs., SD = 1.8 yrs.) TD children (mean age = 10.4 yrs., SD = 1.7 yrs.)	Lifting unfamiliar objects	1 session 81 trials	immediate: after 5 min delayed: after 24 hours	NA	All participants: Grip force rate: object differentiation in the 9th block: blocked > random (p < 0.05) other blocks: not reported Load force rate: object differentiation in the 9th block: blocked > random (p < 0.001) other blocks: no significant group difference Acceleration after lift-off: object differentiation in the 1st block: blocked > random (p < 0.01) Acceleration between objects more similar for blocked vs. random group (p > 0.05) CP: not reported TD: not reported	All participants: no significant group difference CP: not reported TD: not reported	NA
	Total: 36		Tasks themselves	tasks themselves	NA			
	Children with CP: Blocked: 9 Random: 9 TD children: Blocked: 9 Random: 9		Load force rate Acceleration after lift-off Grip force Grip force rate	Load force rate Acceleration after lift-off Grip force Grip force rate	NA			
Edwards et al., 1986 [6]	Children with DS (mean CA = 18.1 yrs., SD = 2.5 yrs.; mean MA = 4.7 yrs., SD = 1.4 yrs.) Children without DS (mean CA = 5.8 yrs., SD = 2.0 yrs.)	Coincident anticipation timing task	1 session 4 x 16 trials	NA	After 10 minutes	Both groups (DS and no DS): Absolute constant error: no significant group difference Variable error: no significant group difference	NA	Inside transfer: Absolute constant error: no significant difference Variable error: significant group x training x block interaction (F_1,36_ = 4.1) random > blocked (no DS) Outside transfer (both groups (DS and no DS): Absolute constant error: no significant difference Variable error: no significant difference
	Total: 40		Task itself	NA	Same task, different speeds (within transfer: speed within the trained speed range; outside transfer: speed outside the trained speed range)			
	Children with DS: 20 Children without DS: 20 *n/practice group unknown*		Absolute constant error Variable error	NA	Absolute constant error Variable error			
Fialho et al., 2006 [33]	Skilled volleyball players (mean age = 16.3 yrs., SD = 0.67 yrs.)	Volleyball skills (tennis serve, float serve)	4 days 1 session / day (46 trials each)	NA	After 10 minutes After 24 hours (retention of the transfer)	Mean score: NA (between group results not reported) SD of the score: no significant group difference	NA	Mean score: Transfer: NA (between group results not reported) Retention of the transfer: random > blocked H_10,1_ = 3.6, P < 0.05) for the first block of trials SD of the score: Transfer: no significant group difference Retention of the transfer: no significant group difference
	Total: 10		Tasks themselves	NA	Asian serve (transfer and retention of the transfer)			
	Blocked: 5 Random: 5		Accuracy scores of the serves (means and SDs)	NA	Accuracy scores of the serves (means and SDs)			
French et al., 1990 [34]	9th grade students, enrolled in physical education (mean age/SD = NA)	Volleyball skills (forearm pass, overhead set, serve)	6 days 1 session / day (30 trials each)	After 2 days	NA	No significant main or interaction effect	No significant main effect	NA
	Total: 139		Tasks themselves	Tasks themselves	NA			
	Blocked: NA Random: NA Random-blocked: NA		Scores for accuracy and force	Scores for accuracy and force	NA			
Gophna et al., 2007 [26]	Children with LD (mean age = 7.1 yrs., SD = 0.25 yrs.) Children without LD (mean age = 7.9 yrs., SD = 0.31 yrs.)	Mirror tracing task (different shapes)	1 session 36 trials	After 1 week	After 1 week	n errors: random > blocked (all participants, p = 0.02) Error time: no significant group difference Total time: no significant group difference	n errors: no significant group difference Error time: no significant group difference Total time: no significant group difference	n errors: no significant group difference Error time: no significant group difference Total time: no significant group difference
	Total: 48		Tasks themselves (3 shapes)	Tasks themselves (3 shapes)	Same task, 2 different shapes			
	Children with LD: 24 Blocked: 12* Random: 12* Children without LD: 24 Blocked: 12* Random: 12*		n errors Error time Total time (to complete the task)	n errors Error time Total time (to complete the task)	n errors Error time Total time (to complete the task)			
Granda Vera & Montilla, 2003 [35]	6-yr.-old children (considered normal and healthy)	Throwing different balls	6 weeks 3 sessions / week (50 minutes each)	After 2 weeks	After 2 weeks	Vertical target: Tennis ball (3 m): random > blocked (F = 7.54, p = 0.008) Feather fly ball (3 m): random > blocked (F = 5.90, p = 0.02) Tennis ball (5 m): no significant group difference Feather fly ball (5 m): no significant group difference Horizontal target: Tennis ball (3 m): no significant group difference Feather fly ball (3 m): no significant group difference Tennis ball (5 m): no significant group difference Feather fly ball (5 m): no significant group difference Total score vertical target: random > blocked (F = 5.68, p = 0.02) Total score horizontal target: no significant group difference Total score all conditions: random > blocked (F = 3.68, p = 0.05)	Random (mean ± SD = 13.0 ± 4.47) > blocked (mean ± SD = 9.29 ± 3.62) (F = 16.26, p = 0.001)	Random (mean ± SD = 10.08 ± 54.01) > blocked (mean ± SD = 7.58 ± 2.72) (F = 11.79, p = 0.001)
	Total: 71		Tasks themselves	Tasks themselves	Same task, different ball, different distance			
	Blocked: 34 Random: 37		Performance score	Performance score	Performance score			
Green et al., 1995 [36]	Female students (first year secondary school eastern England), right handed novices in racket sports	Hitting different balls with different rackets (tennis, squash, badminton, short tennis)	4 days (48 trials each)	After 1 minute, after 1, 4 and 8 days	After 1 minute. after 1, 4 and 8 days	No significant group difference	NA	Both dimensions out of range: (racket and target): random > blocked (F = 6.84, p < 0.05) One dimension out of range: (racket or target): blocked > random (F = 10.07, p < 0.01)
	Total: 48		Tasks themselves	Tasks themselves	Same task, different racket and / or different target			
	Control: 12* Specific: 12* Blocked: 12* Random: 12*		Error scores (target accuracy)	Error scores (target accuracy)	Error scores (target accuracy)			
Jarus & Goreover, 1999 [37]	Healthy children, no motor or cognitive deficits (mean age = 8.15 yrs., SD = 2.44 yrs.)	Throwing beanbags of different sizes to targets of different distances	1 session 30 trials	After 30 minutes	After 30 minutes	All participants: blocked (mean ± SD = 21.30 ± 9.66 cm) > random (mean ± SD = 26.30 ± 12.20 cm) F_1,81_ = 5.29, p < 0.01 5-yr.-old: no significant group difference 7-yr.-old: blocked (mean ± SD = 22.11 ± 8.65 cm) > random (mean ± SD = 33.11 ± 11.17 cm) 11-yr.-old: no significant group difference	All participants: blocked (mean ± SD = 19.75 ± 10.29 cm) > random (mean ± SD = 25.30 ± 11.37 cm) F_3,108_ = 3.73, p < 0.05 5-yr.-old: no significant group difference 7-yr.-old: blocked (mean ± SD = 17.17 ± 8.05 cm) > random (mean ± SD = 28.25 ± 8.34 cm) 11-yr.-old: no significant group difference	No significant group difference for neither all participants nor any of the age groups
	Total: 120		Tasks themselves	Tasks themselves	Same task, different targets			
	5-yr.-old: 40 Blocked: 10 Random 10 Combined:10 Control: 10 7-yr.-old: 40 Blocked: 10 Random 10 Combined:10 Control: 10 11-yr.-old: 40 Blocked: 10 Random 10 Combined:10 Control: 10		Distance from target	Distance from target	Distance from target			
Jarus & Gutman, 2001 [14]	Children from public school, no cognitive and motor deficits (mean age = 8.52 yrs., SD = 0.61 yrs.)	Throwing beanbags of different sizes to targets of different distances Simple task: different bag weights Complex task: different bag weights, and sizes, different target order	1 session 30 trials	After one day	After one day	Simple task: Total time: no significant group difference Accuracy score: not reported due to lack of significant results Complex task: Total time: blocked (mean ± SD = 10.83 ± 3.1) > random (mean ± SD = 14.94 ± 4.34) Accuracy score: not reported due to lack of significant results	Simple task: Total time: no significant group difference Accuracy score: not reported due to lack of significant results Complex task: Total time: no significant group difference Accuracy score: not reported due to lack of significant results	Simple task: Total time: no significant group difference Accuracy score: not reported due to lack of significant results Complex task: Total time: no significant group difference Accuracy score: not reported due to lack of significant results
	Total: 96		Tasks themselves	Tasks themselves	Same task, different bag (simple transfer), different bag and different target order (complex transfer)			
	Simple task: 48 Blocked: 16 Random: 16 Combined: 16 Complex task: 48 Blocked: 16 Random: 16 Combined: 16		Total time to complete each trial Accuracy score (not reported due to lack of significant results)	Total time to complete each trial Accuracy score (not reported due to lack of significant results)	Total time to complete each trial Accuracy score (not reported due to lack of significant results)			
Jones & French, 2007 [38]	9th grade students from 3 high-schools, physical educational classes	Volleyball skills (underhand serve, forearm pass, overhead set)	9 days 30 trials / day (approximately 30 min)	After 2 days	NA	No significant group difference (p>0.05)	No significant group difference (p>0.05)	NA
	Total: 68 51 completed the whole procedure		Tasks themselves	Tasks themselves	NA			
	Blocked: 18* Random: 17* Blocked-random: 16*		Scores (accuracy of the volleyball skills)	Scores (accuracy of the volleyball skills)	NA			
Meira & Tani, 2003 [39]	Female students, secondary school, right-handed, volleyball Novices (mean age = 12.7 yrs., SD = NA)	Volleyball skills (underhand serve, overhand serve, Asian floater)	8 sessions (2 / week) 36 trials / session	NA	Immediately after: Transfer 1 3 sessions (2 / week) 28 trials / session 1 week after: Transfer 2 1 session 12 trials	Precision scores on target: no significant group difference movement pattern quality scores: No significant group difference	NA	No significant group difference in neither transfer 1 nor 2, in neither of the parameters
	Total: 36		Tasks themselves, with knowledge of result	NA	Tasks themselves, without knowledge of result			
	Blocked: 18 Random: 18		Precision scores on target Movement pattern quality scores	NA	Precision scores on target movement pattern quality scores			
Painter et al., 1994 [27]	Students enrolled in IDD classrooms at public junior high-school (mean age = 13.9 yrs., SD = NA) Gender- and CA- matched controls from same school district (mean age = 13.11 yrs., SD = NA)	Throwing beanbags with different throws (underhand throw, overhand throw, hook throw)	1 session 45 trials (3 x 15)	After 10 minutes	NA	Absolute error: IDD: no significant group difference Controls: no significant group difference Combined: significant trial block x schedule interaction (F_14,616_ = 3.84, p < 0.001), significant error reduction in random group but not in blocked group Variable error: IDD: not reported Controls: not reported Combined: not reported	Absolute error: IDD: blocked > random (p < 0.05 for all comparisons) Controls: no significant group difference Combined: random > blocked (F_1,44_ = 15.77, p < 0.001) Variable error: IDD: not reported Controls: not reported Combined: consistency random > blocked (F_1,44_ = 7.94, p < 0.007)	NA
	Total: 48		Tasks themselves	Tasks themselves	NA			
	Children with IDD: 24 Blocked: NA Random: NA Control group: 24 Blocked: NA Random: NA *Equal number of males/females in each practice group*		Absolute error Variable error	Absolute error Variable error	NA			
Perez et al., 2005 [40]	Children (mean age = 10.5 yrs., SD = 0.6 yrs.)	Positioning motor task	1 session 60 trials	NA	Immediate transfer: 10 trials, after 5 minutes Delayed transfer: 20 trials, after 24 hours	Blocked > random (p < 0.01) in all acquisition trials except block 9 (no significant group difference)	NA	Immediate transfer: not reported Delayed transfer: random > blocked (p < 0.0001) for the first block
	Total: 57		Task itself	NA	Task itself, different position, no knowledge of result			
	Blocked: 29 Random: 28		Absolute error	NA	Absolute error			
Pigott & Shapiro, 1984 [7]	Students from the Montessori school in Santa Monica, CA (age range = 6 yrs. 9 months to 8 yrs. 3 months)	Throwing bean bags (different weights)	1 session 24 trials	NA	Immediately after 3 trials	No significant group differences	NA	No significant group differences
	Total: 64		Task itself	NA	Task itself, different weights			
	Blocked: 16 Random: 16 Random-blocked: 16 Constant: 16 *Groups balanced for sex and average age*		Absolute error	NA	Absolute error			
Pollock & Lee, 1997 [41]	Children, 7-yr.-old (mean age = 7.2 yrs., SD = 0.4 yrs.) Adults (mean age = 24.3 yrs., SD = 3.1 yrs.)	Propelling a wooden disk with the middle finger (adaptation of the Crokinole game)	1 session 90 trials (3 x 30)	Immediately after (after transfer test) 15 trials (3 x 5)	Immediately after 2 tests, 10 trials each	All participants: blocked (mean ± SD = 1.4 ± 0.8) > random (mean ± SD = 1.2 ± 0.8) significant main effect for order (F_1,44_ = 5.16, p < 0.05) Children: blocked (mean ± SD = 0.8 ± 0.6) = random (mean±SD = 0.8±0.7) Adults: blocked (mean ± SD = 2.1 ± 1.0) > random (mean ± SD = 1.6 ± 1.0)	All participants: blocked (mean ± SD = 1.2 ± 0.7) < random (mean ± SD = 1.5 ± 0.9) significant main effect for order (F_1,44_ = 4.13, p < 0.05) Children: not reported Adults: not reported	All participants: blocked (mean ± SD = 1.3 ± 0.8) = random (mean ± SD = 1.7 ± 0.9) F_1,44_ = 3.76, p = 0.06 Children: not reported Adults: not reported
	Total: 48		Task itself	Task itself	Task itself, new starting point, new "bumper			
	Children: 24 Blocked: NA Random: NA Adults: 24 Blocked: NA Random: NA *Group membership balanced by sex*		Scores (accuracy of the target)	Scores (accuracy of the target)	Scores (accuracy of the target)			
Saemi et al., 2012 [42]	Male elementary school students, low skilled in throwing tasks (mean age = 10.47 yrs., SD = 0.77 yrs.)	Throwing tennis balls from different starting positions to different targets	1 session 81 trials (3 x 27)	After 1 day (12 trials)	NA	Significant main effect for practice condition (F_2,33_ = 4.19, p < 0.024, η^2^ = 0.203) but no post-hoc pairwise comparison reported No significant practice condition x trial block interaction (F_16,164_ = 0.44, p = 0.97)	No significant group difference	NA
	Total: 36		Task itself	Task itself	NA			
	Blocked: NA Random: NA Increasing: NA		Scores (accuracy of the target)	Scores (accuracy of the target)	NA			
Stambaugh, 2011 [43]	Beginning clarinet players (from 16 elementary schools, 5th or 6th grade, in five school districts from the northwest United States (mean age = NA, SD = NA)	Playing clarinet	3 sessions 18 trials / session	After 24 hours 3 trials	After 24 hours (after retention) 3 trials of each task	Speed: blocked > random (blocks 1, 2, 3) random > blocked (block 4 and further) (F_5,34_ = 5.052, p = 0.001) Accuracy: no significant group difference (p = 0.28) Temporal evenness: no significant group difference (p = 0.35)	Speed: random > blocked (F_1,38_ = 24.953, p < 0.001) Accuracy: no significant group difference (p = 0.44) Temporal evenness: no significant group difference (p = 0.06)	Speed: no significant group difference (p = 0.78) Accuracy: no significant group difference (p = 0.36) Temporal evenness: no significant group difference (p = 0.88)
	Total: 41		Task itself	Task itself	Task itself, other note sequence			
	Blocked: 22 Random: 19		Scores (speed, accuracy, temporal evenness)	Scores (speed, accuracy, temporal evenness)	Scores (speed, accuracy, temporal evenness)			
Ste-Marie et al., 2004 [25] Experiment 1	Children from 3 1st grade classes from 2 Catholic elementary schools (21 females: mean age = 6.3 yrs., SD = 0.43 yrs.; 23 males: mean age = 6.2yrs., SD = 0.55 yrs.)	Handwriting tasks	1 session (35 minutes) 72 trials (24 trials / symbol)	After 30 minutes	NA	No significant group difference blocked (mean ± SD = 2.42 ± 0.95) = random (mean ± SD = 2.26 ± 0.86) (F_1,42_ = 3.2, MSE = 1.9, p = 0.08)	Random > blocked (for both retention groups) (F_1,42_ = 4.2, MSE = 129, p = not reported)	NA
	Total: 44		Tasks themselves	Tasks themselves (either in blocked or random order)	NA			
	Blocked: NA Random: NA		Scores (scoring of the handwritten symbols)	Scores (scoring of the handwritten symbols)	NA			
Ste-Marie et al., 2004 [25] Experiment 2	Children, recruited from 2 schools (mean age = 6.90 yrs., SD = 0.51 yrs.)	Handwriting tasks	1 session (35 minutes) 72 trials	After 20 minutes After 24 hours	After 20 minutes After 24 hours (after the retention test)	Random > blocked (trial sets 2, 3, 4) Blocked = random (trial sets 1, 5, 6, 7, 8) (F_7,322_ = 3.2, MSE = 0.67, p = not reported)	20 minutes retention: significant letter x group interaction (F_2,92_ = 3.4, MSE = 0.090) Blocked: significant writing benefit for letter H Random: significant writing benefit for letter A 24 hours retention: no significant group differences	20 minutes transfer: random (mean ± SD = 23.8 ± 5.7 s) blocked (mean ± SD = 28.3 ± 6.2 s) (F_1,46_ = 4.4, MSE = 165.6) 24 hours transfer: random (mean ± SD = 25.9 ± 4.8 s) > blocked (mean ± SD = 28.2 ± 5.5 s) F_1,46_ = 8.7, MSE = 91.5)
	Total: 50 48 included in analysis		Tasks themselves	Tasks themselves (either in blocked or random order)	Same tasks (same letters but in cursive script continuously)			
	Blocked: 24 Random: 24		Scores (scoring of the handwritten symbols)	Scores (scoring of the handwritten symbols)	Time taken to write the word			
Ste-Marie et al., 2004 [25] Experiment 3	Children, recruited from 5 schools (Montessori, public and Catholic school) (mean age = 6.42 yrs., SD = 0.40 yrs.)	Handwriting tasks (one-to-one-situation, two experimenters, each testing a child)	1 session (35 minutes) 72 trials	After 20 minutes after 24 hours	After 20 minutes after 24 hours (after the retention test)	No significant group difference	20 minutes retention: no significant group difference blocked (mean ± SD = 3.60 ± 0.97) = random (mean ± SD = 4.30 ± 0.83) (p = 0.10) 24 hours retention: no significant group difference	20 minutes transfer: random (mean ± SD = 22.91 ± 7.22 s) > blocked (mean ± SD = 28.41 ± 9.46 s) (F_1,66_ = 7.2, MSE = 69.9, p = not reported) 24 hours transfer: random (mean ± SD = 20.13 ± 6.43 s) > blocked (mean ± SD = 24.76 ± 9.38 s) (F_1,66_ = 5.6, MSE = 63.6, p = not reported)
	Total: 78 68 included in analysis		Tasks themselves	Tasks themselves (either in blocked or random order)	Same tasks (same letters but in cursive script continuously)			
	Blocked: NA Random: NA		Scores (scoring of the handwritten symbols)	Scores (scoring of the handwritten symbols)	Time taken to write the word			
Wegman, 1999 [44]	Female 4th grade students (mean age = NA, SD = NA)	Ball rolling, racket striking, ball kicking	1 session (45 minutes) 39 trials (13 trials / task)	After 3 weeks	NA	Ball rolling: blocked (mean ± SD = 64.41 ± 17.75) > random (mean ± SD = 48.94 ± 17.76) (F_2,53_ = 4.63, p < 0.05) Racket striking: blocked (mean ± SD = 66.47 ± 6.31) > random (mean ± SD = 59.16 ± 11.78) (F_2,53_ = 3.22, p < 0.05) Ball kicking: blocked (mean ± SD = 63.75 ± 6.19) > random (mean ± SD = 52.63 ± 20.77) (F_2,52_ = 3.35, p < 0.05)	Ball rolling: no significant group difference: blocked (mean ± SD = 58.12 ± 16.21) = random (mean ± SD = 55.00 ± 18.85) (F_2,49_ = 0.34, p > 0.05) Racket striking: random (mean ± SD = 74.16 ± 11.91) > blocked (mean ± SD = 54.41 ± 20.45) (F_2,49_ = 4.73, p < 0.05) posttest: F1,31 = 7.41, p < 0.05) Ball kicking: no significant goup difference: blocked (mean ± SD = 50.62 ± 21.74) = random (mean ± SD = 48.68 ± 20.53) (F_2,49_ = 0.24, p > 0.05)	NA
	Total: 54		Tasks themselves	Tasks themselves	NA			
	Repetitions: NA Random: NA Combined: NA		Scores (accuracy)	Scores (accuracy)	NA			
Wrisberg & Mead, 1983 [45]	Right handed children (mean age = 7 yrs., 2 months, SD = NA)	Anticipation timing task (visual tracking of a moving light, different speeds)	2 days 1 session / day 48 trials / day	NA	After one day 20 trials	No significant group effect (F_3,40_ = 0.37, p > 0.05)	NA	Mean absolute error: Slow velocity transfer: blocked (mean ± SD = 97 ± 56) > random (mean ± SD = 146 ± 63) (F_4,50_ = 3.63, p < 0.05) Fast velocity transfer: no significant group difference Mean variable error: no significant group differences Constant error: significant type of training x blocks interaction (F_4,50_ = 2.92, p < 0.05) block 2: late responding significantly different: Varied-random-speed: mean ± SD = 41 ± 83 ms (too late), Varied-blocked-speed: mean ± SD = -19 ± 41 ms (too early)
	Total: 60		Task itself	NA	Task itself, 2 different speeds			
	Slow speed: 12* Fast speed: 12* Varied-random: 12* Varied-blocked: 12* Control: 12* Equal number of females and males		Mean absolute error	NA	Mean absolute error Mean constant error Mean variable error			
Zetou et al., 2007 [46]	Female, unskilled volleyball players (mean age = 12.4 yrs., SD = 1.2)	Volleyball skills (set, pass, service)	10 weeks 2 sessions / week (75 minutes and 72 trials each)	After 2 weeks	NA	Set: no significant group difference (main effect for group: F_1,24_ = 0.11, p = 0.74) Pass: no significant group difference (main effect for group: F_1,24_ = 0.04, p = 0.84) Service: no significant group difference (main effect for group: F_1,24_ = 0.04, p = 0.85)	Set: no significant group difference (main effect for group: F_1,24_ = 0.11, p = 0.74) Pass: no significant group difference (main effect for group: F_1,24_ = 0.04, p = 0.84) Service: no significant group difference (main effect for group: F_1,24_ = 0.04, p = 0.85)	NA
	Total: 26		Tasks themselves	Tasks themselves	NA			
	Low interference: 13 High interference: 13		Scores (ability to play the ball, hitting target)	Scores (ability to play the ball, hitting target)	NA			

Results: between-groups-effects (blocked vs. random practice) were evaluated. If additional groups (e.g. serial, repetitive) were evaluated, these results were not considered. Abbreviations: ACE = absolute constant error; CA = chronological age; CI = contextual interference; CP = cerebral palsy; DS = Down’s Syndrome; IDD = intellectual developmental disability; LD = learning disabilities; m = meters; MA = mental age; ms = milliseconds; MSE = mean square error; n = number; NA = not applicable; SD = standard deviation; TD = typically developing; yr./yrs. = year/years; > meaning "better than".

*n per group is not mentioned in the paper, the information was given by the authors answering to our e-mail.

### The methodological quality of the studies

#### Evidence levels (Table 2)

Most studies have an evidence level II or III, except for one, which had a level I [34]. Eight studies did not perform a randomisation [7,14,31–33,35,37,45], and were rated as level III. Two studies used cluster randomisation of school classes [30,44]. One study randomly divided the participants into a complex and a simple task group and then further subdivided these groups into subgroups [14], but as this latter subdivision was not described, we did not consider it randomisation.

**Table 2:** Levels of evidence and conduct quality.

Study	Evidence level	Quality							
		Conduct questions							
		Summary	1	2	3	4	5	6	7
Bertollo et al., 2010 [29]	II	0/7	no	no	no	no	no	no	no
Bortoli et al., 1992 [30]	II	0/7	no	no	no	no	no	no	no
Broadbent et al., 2015 [31]	III	2/7	no	no	yes	no	no	yes	no
Del Rey et al., 1983 [32]	III	0/7	no	no	no	no	no	no	no
Duff et al., 2003 [28]	II	0/7	no	no	no	no	no	no	no
Edwards et al., 1986 [6]	II	0/7	no	no	no	no	no	no	no
Fialho et al., 2006 [33]	III	0/7	no	no	no	no	no	no	no
French et al., 1990 [34]	I	0/7	no	no	no	no	no	no	no
Gophna et al., 2007 [26]	II	0/7	no	no	no	no	no	no	no
Granda Vera & Montilla, 2003 [35]	III	0/7	no	no	no	no	no	no	no
Green et al., 1995 [36]	II	0/7	no	no	no	no	no	no	no
Jarus & Goreover, 1999 [37]	III	0/7	no	no	no	no	no	no	no
Jarus & Gutman, 2001 [14]	III	0/7	no	no	no	no	no	no	no
Jones & French, 2007 [38]	II	1/7	no	no	no	no	no	yes	no
Meira & Tani, 2003 [39]	II	0/7	no	no	no	no	no	no	no
Painter et al., 1994 [27]	II	0/7	no	no	no	no	no	no	no
Perez et al., 2005 [40]	II	0/7	no	no	no	no	no	no	no
Pigott & Shapiro, 1984 [7]	III	0/7	no	no	no	no	no	no	no
Pollock & Lee, 1997 [41]	II	0/7	no	no	no	no	no	no	no
Saemi et al., 2012 [42]	II	0/7	no	no	no	no	no	no	no
Stambaugh, 2011 [43]	II	0/7	no	no	no	no	no	no	no
Ste-Marie et al., 2004 [25] Experiment 1	II	1/7	no	no	no	yes	no	no	no
Ste-Marie et al., 2004 [25] Experiment 2	II	2/7	no	no	no	yes	no	yes	no
Ste-Marie et al., 2004 [25] Experiment 3	II	2/7	no	no	no	yes	no	yes	no
Wegman, 1999 [44]	II	0/7	no	no	no	no	no	no	no
Wrisberg & Mead, 1983 [45]	III	0/7	no	no	no	no	no	no	no
Zetou et al., 2007 [46]	II	0/7	no	no	no	no	no	no	no

Evidence levels and scoring of the conduct questions of all the included studies. Evidence levels: level I = randomised controlled trials (sample size > 100); level II randomised controlled trials (sample size < 100); level III: controlled cohort studies; level IV: case series; level V: expert opinions [15]. Conduct questions: 1) inclusion and exclusion criteria, 2) description of and adherence to the intervention, 3) validity and reliability of outcome measures, 4) masking of the participants and assessors, 5) statistical analysis, 6) dropouts, 7) controlling for confounding variables.[15]

#### Quality of conduct (Table 2)

The methodological quality of the studies was low. Twenty-two studies received 0 out of 7 points. No study received a point for the questions 1 (allocation, randomisation), 2 (description and adherence of interventions), 5 (statistics), and 7 (appropriate methods the control confounding and bias). Two studies received 1 point (study by Jones & French [38], experiment 1 from Ste-Marie et al. [25]) and three studies received 2 points (study by Broadbent et al. [31], experiments 2 and 3 from Ste-Marie et al. [25]).

#### Risk of bias

Most of the studies had a high risk of bias in all domains, except for Broadbent et al. [31] who defined the primary outcome measure and presented the results for all time points [31]. Therefore, we rated the risk of attrition bias as low.

Combining the study results by pooling the data in a meta-analysis was not appropriate since the studies were too heterogeneous considering the populations, types of motor tasks, intensities, time points (e.g. retention after five minutes, 24 hours or three weeks), and outcome measures. We also refrained from a subgroup analysis due to the low methodological quality and too small sample sizes of studies with sufficient relevant similarities.

### Best evidence synthesis

The best evidence synthesis (Table 3) was conducted for the typically developing children. We grouped the studies according to the tasks they evaluated and received 15 task-specific groups. For most tasks, the evidence was conflicting or absent. Single tasks showed limited to moderate evidence supporting the contextual interference effect. Acquisition: there was limited evidence for the benefit of blocked practice over random practice for dance step sequence [29], ball rolling, striking, and kicking [44], and a positioning motor task [40]. Retention: There was limited evidence for the benefit of random practice over blocked practice for throwing different balls [35] and playing tennis in a simulated environment [31]. Transfer: Moderately consistent evidence was found for the benefit of random practice over blocked practice for handwriting skills [25] and limited evidence for throwing different balls [35].

**Table 3:** Best evidence synthesis according to tasks, learning level and practice order.

Area		Task	Study	Evidence synthesis per study			Evidence synthesis summary		
				Acquisition	Retention	Transfer	Acquisition	Retention	Transfer
NLT	FMT	Mirror tracing task	Gophna et al., 2007 [26]	NS/NR	NS	NS	-	-	-
		Playing clarinet	Stambaugh, 2011[43]	IC	IC	NS	X	X	-
		Handwriting skills	Ste-Marie et al., 2004 [25] Experiment 1	NS	R	NA	X	X	** R
			Ste-Marie et al., 2004 [25] Experiment 2	IC	IC	R			
			Ste-Marie et al., 2004 [25] Experiment 3	NS	NS	R			
	GMT	Throwing beanbags	Jarus & Goreover, 1999 [37]	IC	IC	IC	X	X	X
			Jarus & Gutman, 2001 [14]	IC	NS	NS			
			Painter et al., 1994 [27]	NS/NR	NS/NR	NA			
			Pigott & Shapiro, 1984 [7]	NS	NA	NS			
		Throwing different balls	Granda Vera & Montilla, 2003 [35]	IC	R	R	X	* R	* R
		Throwing tennis balls	Saemi et al., 2012 [42]	NS	NS	NA	-	-	NA
		Volleyball	Bortoli et al., 1992 [30]	NS	NS	IC	-	-	X
			Fialho et al., 2006 [33]	NS/NR	NA	IC			
			French et al., 1990 [34]	NS	NS	NA			
			Jones & French, 2007 [38]	NS	NS	NA			
			Meira & Tani, 2003 [39]	NS	NA	NS			
			Zetou et al., 2007 [46]	NS	NS	NA			
		Hitting different balls with different rackets	Green et al., 1995 [36]	NS	NA	IC	-	NA	X
		Lifting unfamiliar objects	Duff et al., 2003 [28]	NR	NR	NA	-	-	NA
		Dance step sequence	Bertollo et al., 2010 [29]	B	NS	NA	* B	-	NA
		Propelling task	Pollock & Lee, 1997 [41]	NS	NR	NR	-	-	-
		Tennis	Broadbent et al., 2015 [31]	NS	R	IC	-	* R	X
		Ball rolling, striking, kicking	Wegman, 1999 [44]	B	IC	NA	* B	X	NA
LT	FMT	Anticipation timing task	Del Rey et al., 1983 [32]	B	NA	NS	X	NA	X
			Edwards et al., 1986 [6]	NR	NA	IC			
			Wrisberg & Mead, 1983 [45]	NS	NA	IC			
		Positioning motor task	Perez et al., 2005 [40]	B	IC	NA	* B	X	NA

Abbreviations: NLT = Non-laboratory tasks; LT = Laboratory tasks; FMT = Fine-motor tasks GMT = Gross motor tasks; B = significant, favouring blocked order; IC = inconsistent; NA = not applicable, no study evaluated the according aspect; NR = not reported; NS = not significant; R = significant, favouring random order. Evaluation of the studies: Results of the single studies were evaluated taking in account the typically developing children and all parameters and tasks into account. Results with ≥ 75% of the comparisons favouring one practice order were evaluated as consistent evidence within one study. Evaluation of the tasks: Results of the according studies were merged if ≥ 75% of the studies of one task showed the same result, evidence was rated as consistent. Strength of the evidence (adapted from Tulder et al. [24]):

*** = Strong—consistent findings among multiple high quality randomised controlled trials (RCTs)

** = Moderate—consistent findings among multiple low quality RCTs and/or controlled clinical trials (CCTs) and/or high one high quality RCT

* = Limited—one low quality RCT and/or CCT

X = Conflicting—inconsistent findings among multiple trials (RCTs and/or CCTs); inconsistent findings among different parameters within one trial (if only one trial is available)

- = No evidence from trials—no RCTs or CCTs

## Discussion

We investigated the evidence of contextual interference in children with congenital or acquired brain injuries and typically developing children. Only one study included children with brain lesions. The methodological quality of the studies was low and the risk of bias high, which makes it difficult to formulate recommendations whether children with brain lesions or typically developing children would profit more from a blocked or random approach.

### Contextual interference in children with hemiplegic cerebral palsy

The one study examining children with hemiplegic CP (n = 18, mean age 10 years, SD 1.8) also included a group of age-matched typically developing peers (n = 18, mean age 10.4 years, SD 1.7 years) [28].The study consisted of two experiments. In the first experiment, children lifted various known objects while the vertical lifting load force was measured. The second experiment investigated the contextual interference effect. The participants lifted three novel objects with varying weights 27 times. One group did this in blocked order, the other group in random order. Retention was tested immediately after and 24 hours after the practice phase. While during acquisition blocked practice resulted in better differentiation of force rates between the different objects, there was no difference during the retention trials between the practice groups. Based on these two experiments the authors concluded that children with hemiplegic CP have an internal picture of the weight of familiar objects, that they can learn and retain to provide the right amount of force when lifting objects with unknown weights, but that the amount of practice rather than the practice order is essential for this learning process [28]. A conclusion about the contextual interference effect in children with CP is difficult, though, because this was the only study we found and it had some qualitative shortcomings. In the methodological quality assessment, this study received 0 of 7 points (Table 2) and the risk of bias was high. The main reasons for our low rating of this study were the lack of information about the control group at baseline (only the means of the whole groups are reported without a measure of variation), the missing information about the adherence, the psychometric properties of the assessments were not reported, it was unclear whether assessors were masked, the lack of a power calculation, and the number of drop-outs were not reported.

### Contextual interference in typically developing children

When considering contextual interference studies with typically developing children, a conclusion also remains unclear. Although the best evidence synthesis showed limited to moderate support of the contextual interference effect for some of the tasks (favouring random practice for better retention and transfer), in the majority of the tasks no evidence (acquisition: n = 7/15, retention: 6/13, transfer: n = 3/10) or conflicting evidence (acquisition: n = 5/15, retention: n = 5/13, transfer: n = 5/10) was found (Table 3). Besides the low methodological quality, several factors could have affected the contextual interference effect contributing to the inconclusiveness of some results [2].

### The influence of types of skills and variations

One of these factors might be the kind of skill and its variations that were studied. For example, Magill and Hall already discussed that the generalisability of the contextual interference effect could be influenced by task characteristics like laboratory tasks, such as coincident anticipation timing tasks, versus motor skill performance outside the laboratory or non-laboratory tasks, such as throwing beanbags [5].

In our review, we found four studies that investigated laboratory tasks, namely anticipation timing tasks [6,32,45], and a positioning motor task [40]. The other studies investigated non-laboratory tasks: six examined volleyball skills [30,33,34,38,39,46], four beanbag throwing [7,14,27,37], and three experiments investigated handwriting skills [25]. Further tasks were dance step sequences [29], tennis skills [31], lifting unfamiliar objects [28], mirror tracing tasks [26], throwing different balls [35], hitting different balls with different rackets [36], rolling, striking and hitting balls [44], a propelling task (Crokinole game) [41], throwing tennis balls [42], and playing the clarinet [43].

From a therapeutic point of view, this change of interest from laboratory to non-laboratory tasks is desired. While learning a laboratory task can reflect the capacity of the child, i.e. what a person with a health condition actually is able to do, learning non-laboratory tasks may better resemble daily life activities, i.e. performance (what a person does in his/her usual environment, e.g. skills or tasks needed for self-care, leisure activities, school or work), as described by the World Health Organisation’s International Classification of Functioning, Disability and Health (ICF) [47]. Practicing non-laboratory tasks might improve the translation to other daily life relevant tasks, as these tasks might appear more natural and are probably more frequently occurring in the child’s daily routines than laboratory tasks.

In healthy adults, the evidence is mixed when practicing laboratory tasks, but practicing non-laboratory tasks supports the contextual interference effect [5]. In our review, the best evidence synthesis shows limited to moderate support for the contextual interference effect in five non-laboratory and one laboratory task (Table 3). However, only in a “throwing different balls” [35] task, we found the contextual interference effect for both the retention and transfer. In summary, a clear differentiation about how laboratory or non-laboratory tasks support the contextual interference effect in children cannot be determined.

### The influence of experience, age and task difficulty

In adults, it is suggested that the learner needs to have an idea of the movement or some initial experience to profit from variations of the practice schedule, but the relation between age and the contextual interference remains unclear [5]. In children, Jarus and Goreover observed a difference between three age groups (5 years, 7 years, and 11 years) who practiced beanbag throwing [37]. In general, older children performed the bean bag throwing task better. While the group of 7-year-old children acquired and retained better during blocked practice, there was no difference between the practice groups during the transfer [37]. Hence, for this task, blocked practice might be more beneficial for this age group. While the authors argued that this finding could be explained by the low experience level and the young age of the 7-year-old children, these observations were not made for the 5-year-old children, which is not in line with the author’s explanation. Furthermore, Pollock and Lee compared the learning of propelling a small wooden disk with the middle finger (an adaptation of the Crokinole game) between children and adults [41]. They could reconstruct the contextual interference effect in adults and also in children during transfer and retention, but the children showed no difference in task acquisition between the blocked and random groups [41]. Apparently, this pattern has been observed in adults practicing a difficult task and can be explained with a benefit of blocked practice during acquisition of easy tasks only [2,48].

If the difficulty level of a task seems to influence only the acquisition but not the retention and transfer in adults, the random practice order can be recommended in adults, regardless whether the task is simple or difficult. In typically developing children, though, we cannot make such a recommendation, because the evidence is unclear as experience, age, and task difficulty intermingle with each other. It becomes even more complicated when trying to generalise the effects of experience, age, and task difficulty on the contextual interference effect to children with congenital or acquired brain lesions. For example, the question of whether children in neurorehabilitation are novices or whether they are experienced has to be considered carefully. Given that children with congenital brain lesions or acquired brain lesions in a chronic state have had therapy for most of their lives or for a long time, respectively, they could be considered an expert group. Children with a (sub-) acute acquired brain injury most likely could, on the one hand, be regarded as novices when it comes to relearning motor activities of daily life with their impaired sensorimotor and cognitive systems. On the other hand, they might have performed all these activities independently before they experienced the brain injury which puts them on another starting point compared to children with congenital brain lesions. All these aspects need to be incorporated when considering the contextual interference effect in paediatric neurorehabilitation.

### Can knowledge about related populations be transferred to children with brain lesions?

The evidence about the contextual interference in typically developing children is limited, yet, more extensive compared to the evidence available for children with brain lesions. In adults with brain lesions, the results are not clearly supporting the contextual interference effect. In adults with chronic stroke, for example, the typical contextual interference effect could not be reconstructed when performing three specific movements (wrist/finger extension, elbow joint extension, and shoulder joint abduction) combined with active neuromuscular stimulation [49]. When practicing a task that was designed to approximate the steps needed take a coffee cup out of the cupboard and put it on the table, the random practice outperformed the blocked group in stroke patients [50]. Schweighofer et al. [51] concluded that these conflicting results might be due to the lack of separation of the patients between high and low working memory capabilities. In their study, individuals with stroke with normal visuospatial working memory retained visuomotor skills better when practicing in random order compared to blocked order, while in participants with low visuospatial working memory retention performance did not differ between practice groups [51]. It seems that also in adults with brain injury results might be influenced by other factors additional to the practice order.

We assume that results obtained in typically developing children (compared to healthy adults) could be better generalised to children with brain lesions. Nevertheless, we should be cautious, because, firstly, the physical requirements are different: damaged sensory pathways and structures involved in processing sensory information, such as found in children with brain lesions, reduce the ability to detect errors and consequently impair motor learning [52,53]. Secondly, learning a motor task with a damaged brain is likely different from learning with an intact, typically developing brain. This stands in contrast with previous observations in adults with a unilateral stroke which suggested that the stroke affected the control and execution, but not the learning of motor skills per se [54]. However, as this issue is under debate, there is still no definite agreement which brain regions and processes are involved in learning and how the learning processes are executed (e.g. [55–57]. Furthermore, the individual lesion areas in combination with many other factors make every patient and their learning abilities and strategies unique. Therefore, further research is needed to understand the relation of pathological changes and motor learning disorders [58]. Thirdly, it has been shown that physiotherapists perceive primary impairments (e.g. muscle tone, movement patterns) and secondary outcomes (e.g. range of motion, joint alignment, muscle strength), as well as personal factors (e.g. motivation) and environmental factors (e.g. support and expectations from the family) as important factors influencing the acquisition of motor abilities in children with CP [59]. These factors could slow down or even hinder learning in children with brain lesions compared to typically developing children.

### The methodological quality of the studies

A reconstruction of the methodological approach was challenging in many studies. It was often unclear whether certain methodological aspects were poorly performed or just poorly described. This influenced our assessment of bias and quality. For example, the psychometric properties of the applied outcome measures were unknown or not reported. Several studies mentioned reliability evaluations of their measures, while information on validity or absolute measurements errors (such as the standard error of measurement) was missing. Also, the description of the appropriate statistics and power calculation (both are needed to score a “yes”) was missing. Only one study mentioned a power analysis but did not present it [37].

**Fig 2:**
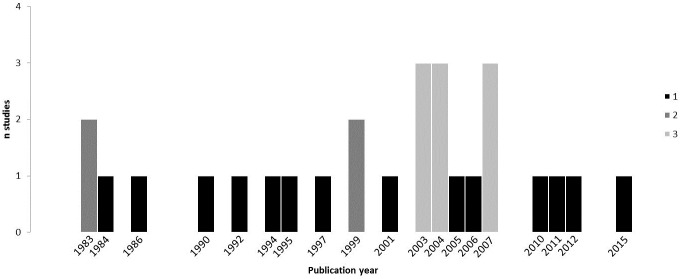
The distribution of the publication years of the articles included in this systematic review.

The oldest papers we included in this review were published in the 1980ies (Fig 2). The Standardised Reporting of Trials (SORT) Statement [60] was published in 1994. Before that checklists for interventional trials were not available. This might partly explain why the older studies did not report all aspects systematically and were therewith rated low in the quality evaluation.

### Limitations

There are some limitations of this systematic review which need to be mentioned. Our literature search was limited to seven databases and restricted to published articles only. Grey literature was not considered. We excluded studies which performed a proceeding familiarisation phase prior to the actual practice phase, whether or not this affects the learning remains to be discussed. For the best evidence synthesis, we did not subdivide the study results according to long- or short-term learning phases because there were not enough comparable studies to build subgroups. This asks for a cautious interpretation of the results.

### Recommendations for future research

We expect that the contextual interference effect in children with brain lesions can influence rehabilitation outcomes. Therefore, we would recommend to design such studies and include these particular patient groups. A careful selection of the motor task to be studied is crucial: it should be clinically relevant and motivating for the child to perform and it should provide objective parameters to quantify the retention or transfer of the task or skill particularly, as these are most relevant for the child after discharge from rehabilitation. The study should be designed and its results reported in accordance with the various internationally accepted checklists to ensure high study quality and low bias.

## Conclusion

To recapitulate, there is a persistent demand for increasing our knowledge about the contextual interference effect in children, especially, in children with brain lesions, as the number of existing studies is small, and the methodological quality of the studies is low. For some tasks, we found limited evidence supporting the contextual interference effect in typically developing children. However, we would be cautious in generalising these results to children with brain lesions. To improve movement or sports programmes in typically developing children and advance rehabilitation programmes for children with brain lesions, there is an emerging need to increase our knowledge of the contextual interference effect in these populations.

## Supporting information

S1 Table Example of the search strategy used for the primary search on PsycINFO. (DOCX)

S2 Table Levels of evidence. Levels of evidence in intervention or group studies and single-case design studies according to the American Academy for Cerebral Palsy and Developmental Medicine (AACPDM) [15]. Abbreviations: ATD = alternating treatment design; MB = multiple baseline; MBD = multiple baseline design; n = number; RCT = randomised controlled trial; SSRD = single subject research design.
(DOCX)

S3 Table Conduct questions. Questions to evaluate the methodology of an intervention study according to the American Academy for Cerebral Palsy and Developmental Medicine [15].
(DOCX)

S4 Table The Cochrane Collaboration’s tool for assessing the risk of bias. The risk of bias tool, presented by the Cochrane handbook for systematic reviews of interventions [16].
(DOCX)

S5 Table PRISMA checklist. (DOC)
