# Supplementary material for: The role of the practice order: A systematic review about contextual interference in children
Source: PLoS One. 2019 Jan 22;14(1):e0209979. doi: 10.1371/journal.pone.0209979 (PMC6342307; doi:10.1371/journal.pone.0209979)
Supplement: S1 Table — (DOCX) [file pone.0209979.s001.docx]

| On PsycINFO we conducted 4 searches with different filters and combined them.  **Basic search term (for all the 4 searches):**   \| ('paediatrics' OR 'pediatrics' OR 'children' OR 'child' OR 'childhood' OR 'adolescent' OR 'adolescence' OR 'student' OR 'elementary' OR 'high school' OR 'youths') \| \| --- \| \| AND ('motor learning' OR 'skill learning') \| \| AND ('contextual interference' OR 'practice order' OR 'random' OR 'blocked') \| \| AND ('performance' OR 'acquisition' OR 'retention' OR 'transfer' OR 'generalisation' OR 'generalization') \|   **Filters:**  Filter for search ♯1: childhood (birth-12 yrs)  Filter for search ♯2: school age (6-12 yrs)  Filter for search ♯3: preschool age (2-5 yrs)  Filter for search ♯4: adolescence (13-17 yrs)  **Combined search:**  ♯1 OR ♯2 OR ♯3 OR ♯4 |
| --- | --- | --- | --- | --- |
