# Supplementary material for: The role of the practice order: A systematic review about contextual interference in children
Source: PLoS One. 2019 Jan 22;14(1):e0209979. doi: 10.1371/journal.pone.0209979 (PMC6342307; doi:10.1371/journal.pone.0209979)
Supplement: S2 Table — Levels of evidence in intervention or group studies and single-case design studies according to the American Academy for Cerebral Palsy and Developmental Medicine (AACPDM) [15]. Abbreviations: ATD = alternating treatment design; MB = multiple baseline; MBD = multiple baseline design; n = number; RCT = randomised controlled trial; SSRD = single subject research design. (DOCX) [file pone.0209979.s002.docx]

| **Level** | **Intervention/Group studies** | **Single subject design studies** |
| --- | --- | --- |
| **I** | Systematic review of randomised controlled trials (RCTs) Large RCT (with narrow confidence interval) (n>100) | Randomised controlled N-of 1 (RCT), alternating  treatment design (ATD), and concurrent or non-concurrent multiple baseline designs (MBDs); generalisability if the ATD is replicated across three or more subjects and the MB consists of a minimum of three subjects, behaviours, or settings. These designs can provide causal inferences. |
| **II** | Smaller RCTs (with wider confidence intervals) (n<100) Systematic reviews of cohort studies Outcome research (very large ecologic studies) | Non-randomised, controlled, concurrent MBD; generalisability if design consists of a minimum of tree subjects, behaviours, or settings. Limited causal inferences. |
| **III** | Cohort studies (must have concurrent control group) Systematic reviews of case control studies | Non-randomised, non-concurrent, controlled MBD; generalisability if design consists of a minimum of three subjects, behaviours or settings. Limited causal inferences. |
| **IV** | Case series Cohort studies without concurrent control group (e.g. with historical control group) Case-control study | Non-randomised, controlled SSRDs with at least three phrases (ABA, ABAB, BAB, etc.); generalisability if replicated across three or more different subjects. Only hints at causal inferences. |
| **V** | Expert opinion Case study or report Bench research Expert opinion based on theory or physiological research Common sense/anecdotes | Non-randomised controlled AB SSRD; generalisability if replicated across three or more different subjects. Suggests causal inferences allowing for testing of ideas. |
