# Supplementary material for: The role of the practice order: A systematic review about contextual interference in children
Source: PLoS One. 2019 Jan 22;14(1):e0209979. doi: 10.1371/journal.pone.0209979 (PMC6342307; doi:10.1371/journal.pone.0209979)
Supplement: S3 Table — Questions to evaluate the methodology of an intervention study according to the American Academy for Cerebral Palsy and Developmental Medicine [15]. (DOCX) [file pone.0209979.s003.docx]

| **No** | **Question** |  |
| --- | --- | --- |
| 1 | Were inclusion and exclusion criteria of the study population well described and followed? |  |
| 2 | Was the intervention well described and was there adherence to the intervention assignment? (For 2 group-designs, was the control exposure also well described?) Both parts of the question need to be met to score "yes". |  |
| 3 | Were all the measures used clearly described, valid and reliable for measuring the outcomes of interest? |  |
| 4 | Was the outcome assessor unaware of the intervention status of the participants (i.e. were the assessors masked?) |  |
| 5 | Did the authors conduct and report appropriate statistical evaluation including power calculations? Both parts of the question need to be met to score "yes". |  |
| 6 | Were dropouts/loss to follow-up reported as less than 20%? For 2-group designs, was dropout balanced? |  |
| 7 | Considering the potential within the study design, were appropriate methods for controlling confounding variables and limiting potential biases used? |  |
|  | | |
