# Supplementary material for: The role of the practice order: A systematic review about contextual interference in children
Source: PLoS One. 2019 Jan 22;14(1):e0209979. doi: 10.1371/journal.pone.0209979 (PMC6342307; doi:10.1371/journal.pone.0209979)
Supplement: S4 Table — The risk of bias tool, presented by the Cochrane handbook for systematic reviews of interventions [16]. (DOCX) [file pone.0209979.s004.docx]

| **Domain** | **Review authors’ judgement** |
| --- | --- |
| ***Selection bias*** | |
| **Random sequence generation** | Selection bias (biased allocation to interventions) due to inadequate generation of a randomised sequence. |
| **Allocation concealment** | Selection bias (biased allocation to interventions) due to inadequate concealment of allocations prior to assignment. |
| ***Performance bias*** | |
| **Blinding of participants and personnel** | Performance bias due to knowledge of the allocated interventions by participants and personnel during the study. *Assessments should be made for each main outcome (or class of outcomes).* |
| ***Detection bias*** | |
| **Blinding of outcome assessment** | Detection bias due to knowledge of the allocated interventions by outcome assessors. *Assessments should be made for each main outcome (or class of outcomes).* |
| ***Attrition bias.*** | |
| **Incomplete outcome data** | Attrition bias due to amount, nature or handling of incomplete outcome data. *Assessments should be made for each main outcome (or class of outcomes).* |
| ***Reporting bias*** | |
| **Selective reporting** | Reporting bias due to selective outcome reporting. |
| ***Other bias*** | |
| **Other sources of bias** | Bias due to problems not covered elsewhere in the table. |
